# Perception of the Food Environment and Availability of Unprocessed and Ultra-Processed Foods in the Households of Brazilian Schoolchildren During the COVID-19 Pandemic

**DOI:** 10.3390/ijerph23030373

**Published:** 2026-03-16

**Authors:** Irene Carolina Sousa Justiniano, Raquel de Deus Mendonça, Priscila Pena Camargo, Adriana Lúcia Meireles, Mariana Carvalho Menezes

**Affiliations:** 1 Postgraduate Programme in Health and Nutrition, Federal University of Ouro Preto, Ouro Preto 35400-000, Brazil; 2 Department of Clinical and Social Nutrition, School of Nutrition, Federal University of Ouro Preto, Ouro Preto 35400-000, Brazil; 3 Department of Nutrition, Federal University of Minas Gerais, Belo Horizonte 30130-100, Brazil

**Keywords:** unprocessed foods, fruits and vegetables, ultra-processed foods, food environment, COVID-19

## Abstract

This study assessed perceptions of the food environment and its association with the availability of unprocessed, minimally processed, and ultra-processed foods in the households of Brazilian schoolchildren during the COVID-19 pandemic. A cross-sectional telephone survey (n = 475) was conducted between March, April and May 2021 with a representative sample of households with public school students from two Brazilian municipalities. Household food availability was assessed using a frequency questionnaire referring to the 30 days prior to the survey. Perception of the food environment was assessed using questions that measured perceived availability, price, and quality of fruits and vegetables (FV) and ultra-processed foods (UPF) sold in the neighbourhood. To analyse the association between perceived food environment and food availability, univariate and multivariate logistic regression analyses were performed, with 95% CI. The results indicate that high availability of unprocessed or minimally processed foods was found in 7.4% of households and high availability of UPF in 92.6%. Positive perception of UPF variety in the neighbourhood was more prevalent in households with greater availability of these foods (*p* < 0.05). After adjustment for sociodemographic characteristics, a positive perception of FV variety was associated with lower odds of high household UPF availability (OR = 0.54; 95%CI: 0.30–0.97). Perception of the food environment is an important factor associated with household UPF availability. Policy interventions should consider promoting healthier food environments by expanding the distribution of fresh foods alongside measures that ensure economic access to these foods.

## 1. Introduction

The COVID-19 pandemic intensified concerns about food availability and access, especially among socially vulnerable populations with reduced purchasing capacity and limited access to unprocessed and minimally processed foods. This scenario undermined the Human Right to Adequate and Healthy Food (HRAHF) and contributed to the worsening of food and nutrition insecurity in several countries, including Brazil [1,2,3,4].

During the pandemic, rising prices of unprocessed and minimally processed foods, combined with income losses or reductions, led many families to adopt less healthy dietary habits, including increased consumption of ultra-processed foods (UPF), which are often more affordable but less nutritious [5]. Additionally, school closures disrupted the regular provision of healthy meals to millions of children and adolescents, placing full responsibility for food provision on families during a period of financial instability [6].

In Brazil, particularly in Minas Gerais, a study conducted during the COVID-19 pandemic reported that the interruption of school meal provision exposed students to a high risk of food insecurity: in the investigated sample, 82% of schoolchildren’s families experienced food insecurity, with 16.3% at moderate or severe levels. Moreover, substantial reductions in household income were significantly associated with food insecurity [7].

Socioeconomic factors such as living conditions, the number and age of household members, sex of the household head, educational attainment, and income are important determinants of inequalities in access to food [8,9]. Thus, financial constraints and contexts of social vulnerability may influence both the acquisition and consumption of foods, particularly UPF, due to their lower cost compared with healthier foods such as fruits and vegetables (FV) [8,10,11].

In addition to socioeconomic factors, characteristics of the food environment—such as availability, price, and location of food outlets—also influence individual dietary behaviours by determining opportunities or barriers for the purchasing and consumption of healthier or less healthy foods [12,13,14,15,16]. Perceptions of the food environment are also relevant, although subjective, and vary according to personal experience, preferences, and social and cultural conditions [16]. Understanding how the perceived food environment relates to household food availability is therefore essential for identifying inequalities in access.

This study aimed to assess perceptions of the food environment during the COVID-19 pandemic and their relationship with the availability of unprocessed and minimally processed foods and ultra-processed foods in the households of Brazilian schoolchildren. This is one of the first studies to examine this association among households of children and adolescents in medium-sized Brazilian municipalities during the pandemic, providing new evidence on the interactions between the community and household food environments. The study used random and stratified sampling to represent the population of public school students in the two municipalities analysed.

## 2. Methods

### 2.1. Study Design and Location

This is a cross-sectional study using data from the Longitudinal Study of Food and Nutrition Security during the COVID-19 Pandemic (ESANP), conducted in two medium-sized Brazilian municipalities (Ouro Preto and Mariana), located in the state of Minas Gerais [7].

Ouro Preto has approximately 74.824 inhabitants, a Human Development Index of 0.741, and an average monthly income of 3.1 minimum wages. Mariana has about 64,830 inhabitants, a Human Development Index of 0.742, and an average monthly income of 2.4 minimum wages [17].

The municipalities were selected because they host the university where the research was conducted and because no previous studies had assessed food security in the school community of the region, nor had the food environments of these municipalities been explored.

### 2.2. Sample and Data Collection

According to school census data, in 2020, 13,219 students were enrolled in municipal public schools in both cities, all of whom were eligible for inclusion. Eligibility was defined as being enrolled in municipal early childhood education or basic education institutions, including nurseries, pre-schools, and primary schools, aged between 6 months and 17 years [7].

Sample size was calculated assuming a 50% prevalence (maximum variability), a 5% margin of error and a 95% confidence level, considering the finite population of 13,219 students. Stratified random sampling with proportional allocation was performed according to educational level (nurseries, pre-schools, and primary schools). This resulted in a minimum required sample of 374 individuals across both cities, proportionally allocated by educational level (nurseries, pre-schools, and primary schools). As the broader study has a longitudinal design, an additional 50% was added to account for potential follow-up losses. The sampling unit was the enrolled student. The data presented in this study were collected in March, April, and May 2021.

The Municipal Education Departments provided lists of students, their guardians, and telephone numbers. Telephone interviews were conducted with the adults responsible for purchasing and/or preparing food in the students’ households. Although the sampling unit was the student, information was obtained from the adult responsible for food purchasing and/or preparation in the household. Five call attempts were made at different times and days for each selected number; unsuccessful contacts were excluded and replaced by new random selections until the sample size was reached.

The study questionnaire included items on sociodemographic characteristics, household food availability, perception of the food environment, and the COVID-19 pandemic.

### 2.3. Outcome: Household Food Availability

The outcome variable was household food availability (domestic food environment) in the 30 days preceding the interview, assessed through the question: “In the last 30 days, did you have [food] at home?” The food list included items commonly consumed by the Brazilian population [18,19] and regionally in the two municipalities: fruits; vegetables; rice/pasta; canjiquinha; beans; milk and dairy products; meat (beef or chicken); eggs; processed meats (mortadella, salami, sausage, ham); canned sardines; frozen foods (chips, pizza, or nuggets); packaged biscuits (cream cracker); packaged snacks (crisps and chips, such as Ruffles^®^, Cheetos^®^, Fandangos^®^); sweets (chocolate, candies, or confectionery); soft drinks; industrialised juices (carton, bottle, powdered); wheat/cassava/corn flour; vegetable oils (soy, corn, coconut, sunflower); lard/butter; margarine; sugar; salt; ready-made seasoning (beef, chicken, or vegetable stock); sliced bread/cake; condensed milk/cream; instant noodles; and ready-made tomato sauce [20].

For each food item, respondents reported the frequency of household availability according to five categories: (i) never (meaning that not even once was the food available); (ii) rarely (meaning that the food was available from time to time); (iii) sometimes (meaning that the food was available sometimes); (iv) almost always (meaning that the food was available often); (v) always (meaning that the food was available every day).

Foods were grouped according to the NOVA classification into: (i) unprocessed or minimally processed foods (NOVA Group 1), which included fruits and vegetables (FV), rice/pasta, canjiquinha, beans, milk and dairy products, meat, eggs, flour, and sardines; and (ii) ultra-processed foods (NOVA Group 4), which included processed meats, frozen foods, packaged biscuits, packaged snacks, sweets, soft drinks, industrialised juices, margarine, ready-made seasoning, sliced bread/cake, condensed milk/cream, instant noodles, and ready-made tomato sauce. Foods belonging to NOVA Group 2 (processed culinary ingredients), such as vegetable oils, lard/butter, sugar, and salt, were excluded from the analysis as these items are primarily used in food preparation rather than consumed directly. Based on availability frequency, “almost always” and “always” were classified as high availability, whereas “never”, “rarely”, and “sometimes” were classified as low availability. This dichotomisation was based on a previous study conducted with the same population and municipalities and was further supported by the low frequencies observed in intermediate categories, which could compromise model stability [20].

### 2.4. Explanatory Variables: Perception of the Food Environment

To assess perception of the neighbourhood food environment, we used questions adapted from a national study [21] that were based on the Nutrition Environment Measures Survey Perceived (NEMS-P), an instrument validated for use in Brazil [22], including statements on perceived access to fruits and vegetables (FV) and ultra-processed foods (UPF): “It is easy to buy fruits and vegetables in your neighbourhood”; “Fruits and vegetables are of good quality in your neighbourhood”; “There is a wide variety of fruits and vegetables in your neighbourhood”; “Fruits and vegetables are cheap in your neighbourhood”; “It is easy to buy soft drinks, cookies, packaged snacks, sweets and other treats in your neighbourhood”; “There is a wide variety of soft drinks, cookies, packaged snacks, sweets and other treats in your neighbourhood”.

These statements represent unprocessed foods (fruits and vegetables) and ultra-processed foods (soft drinks, cookies, packaged snacks, sweets and other treats), according to the NOVA classification and the Brazilian Dietary Guidelines [23,24]. Respondents indicated their level of agreement on a five-point Likert scale ranging from “strongly disagree” to “strongly agree”. For analysis, responses were dichotomised into “agree” (agree and strongly agree) and “do not agree” (neutral, disagree, and strongly disagree) due to small cell counts in some categories.

### 2.5. Covariables

Sociodemographic characteristics included information related to the head of the household (i.e., the person with the highest income): sex (male/female), educational level (elementary, high school, university), marital status (married/not married), age (18–35 years/36–45 years/46–60 years/61 or older), and skin colour (black, including brown and black; or white). Categories such as Asian and Indigenous had no responses in the sample.

Household characteristics included income (minimum wage at the time was US$ 207.27; categories: up to half a minimum wage/1–2 minimum wages/3 or more minimum wages), receipt of social benefits (yes/no), number of adults in the household (1/2/3 or more), and number of children (1/2/3 or more).

COVID-19 related variables included receipt of emergency financial aid (yes/no), decrease in family income during the pandemic (yes/no), and perceived impact of income loss (none/small—including very small and small; medium; large—including large and very large).

### 2.6. Data Analysis

Data were analysed using Stata 14.2. Absolute and relative frequencies (%) and 95% confidence intervals (95%CI) were used to describe variables by food availability level (low/high). Pearson’s chi-square test was used to compare distributions at a 5% significance level. Perceptions of the food environment were described for the overall sample and stratified by household availability of unprocessed and minimally processed foods and ultra-processed foods (low/high).

To examine the association between food environment perception (explanatory variable) and household food availability (outcome: low/high), univariate and multivariate logistic regression analyses were performed, considering a binary outcome. Odds ratios (OR) and 95% confidence intervals (95%CI) were estimated (*p* < 0.05). Multivariate models were adjusted for sociodemographic factors associated with household food availability in descriptive analyses (*p* < 0.05) and supported by theoretical relevance: decrease in family income during the pandemic, sex and educational level of the head of household, marital status, participation in social programmes, receipt of emergency aid, and number of children in the household. All covariates were entered simultaneously in the multivariable models. Multicollinearity among covariates was assessed using variance inflation factors (VIF), and no evidence of problematic collinearity was observed. These variables were conceptualised as potential confounders, as they may independently influence both food environment perception and household food availability, rather than as mediators of the relationship between them. All analyses were conducted using STATA 18.0 statistical software.

This study was approved by the Ethics Committee of the Federal University of Ouro Preto (CAAE: 32005120.6.0000.5150). Verbal informed consent was obtained from all participants prior to data collection. Participants were informed about the study’s purpose, procedures, potential risks and benefits, and their right to withdraw at any time without penalty. Consent was recorded in MP4 format by the interviewer, in accordance with ethical approval.

## 3. Results

A total of 475 households were assessed. High availability of unprocessed or minimally processed foods (NOVA Group 1) was found in 7.4% (n = 35) of households, and high availability of ultra-processed foods (NOVA Group 4) was observed in 92.6% (n = 440). These proportions are not mutually exclusive, as a household could have high availability of both food groups simultaneously; indeed, all households with high availability of NOVA Group 1 foods also had high availability of NOVA Group 4 foods.

The distribution of sociodemographic characteristics according to household food availability is presented in Table 1. Low availability of unprocessed and minimally processed foods (n = 440) and UPF (n = 61) was more prevalent in households headed by women (50.6% and 62.3%, respectively), with heads of household who had completed upper secondary education (60.3% and 55.7%, respectively), with an income of 1 to 2 minimum wages (64.3% and 62.3%, respectively), and who experienced a large reduction in income during the COVID-19 pandemic (39.5% and 59.1%, respectively). In contrast, high availability of unprocessed and minimally processed foods (n = 35) was more frequent in households in which the head of household was married (72.3%), had an income of three minimum wages or more (32.5%), had not received emergency financial aid (85.6%), and had not experienced a decrease in family income during the pandemic (53.0%) (Table 1).

Regarding respondents’ perceptions of the food environment, overall, 64% found it easy to buy FV in the neighbourhood, 67.6% considered these foods of good quality, 57.9% agreed that there was a wide variety of these foods, and 20.6% considered them inexpensive. Regarding UPF, 85.9% of respondents had a positive perception of the ease of buying them in the neighbourhood, and 78.9% agreed that a wide variety of these products was available (Table 2).

Regarding perceptions of the food environment and its association with household food availability, statistically significant associations were observed only for ultra-processed food availability (Table 2). Among respondents who agreed that it is easy to buy FV in the neighbourhood, 65.9% had high availability of UPF (*p* = 0.022). A positive perception of FV quality in the neighbourhood was more prevalent among households with high UPF availability (69.5% vs. 54.1%; *p* = 0.016). Similarly, a positive perception of FV variety in the neighbourhood was more frequent in households with high UPF availability (60.5% vs. 40.9%; *p* = 0.004). Finally, a positive perception of UPF variety in the neighbourhood was more prevalent in households with high availability of these foods (80.4% vs. 68.9%; *p* = 0.038).

In the univariate analysis, results indicated that positive perceptions of the food environment were associated with household UPF availability. For example, the perception of ease in buying FV was associated with lower odds of high UPF availability (OR = 0.53; 95% CI: 0.31–0.91). Similarly, considering FV in the neighbourhood to be of good quality was associated with lower odds of high UPF availability (OR = 0.51; 95%CI: 0.29–0.88), and perceiving a wide variety of FV was associated with lower odds of UPF availability (OR = 0.45; 95%CI: 0.26–0.78). Conversely, the perception of a wide variety of UPF in the neighbourhood was associated with higher odds of high UPF availability at home (OR = 1.85; 95% CI: 1.02–3.36) (Table 3).

In the multivariate analysis, after adjustment for sociodemographic characteristics, the perception of ease in buying FV (OR = 0.65; 95%CI: 0.34–1.09), FV quality (OR = 0.63; 95%CI: 0.35–1.12), FV price (OR = 0.77; 95%CI: 0.35–1.71), ease of buying UPF (OR = 1.84; 95%CI: 0.89–3.82), and variety of UPF (OR = 1.54; 95%CI: 0.81–2.93) were not statistically significant. Only the perception of a wide variety of FV in the neighbourhood remained statistically significant, associated with lower odds of high UPF availability (OR = 0.54; 95%CI: 0.30–0.97). Results for all covariates included in the adjusted models are presented in Table 3.

## 4. Discussion

The results of this study show that, overall, more than half of respondents reported ease of access to FV, whereas ease of access to UPF showed a higher prevalence (approximately 86%). Among individuals with a positive perception of the food environment (particularly regarding a greater variety of FV), a lower likelihood of high household UPF availability was observed. Conversely, the perception of a wide variety of UPF in the neighbourhood was associated with a higher likelihood of households having these foods available.

How individuals perceive their surrounding food environment may be associated with healthy eating practices within the home, though the directionality of this relationship remains uncertain [14,15]. Multiple factors, such as household income, physical access, and lack of information about food and nutrition, influence price perception, food acquisition, and consumption, especially of healthy foods [25].

Evidence from studies conducted in different contexts shows that characteristics of the food environment, such as the availability, accessibility, quality, and price of food, can be associated with the consumption of FV and UPF [26,27,28,29,30]. A cohort study involving 1962 participants across 4 Caribbean countries found that a better perception of the availability and quality of FV was associated with higher consumption of these foods [31]. The present findings suggest that a positive perception of FV variety in the neighbourhood was associated with lower odds of high UPF availability in the household. Although counterintuitive at first glance, this finding is consistent with a substitution hypothesis: households that perceive their neighbourhood as having good access to FV may be more likely to acquire these foods, potentially displacing UPF from the household food supply. This interpretation should be treated with caution, however, given the cross-sectional design of the study, which precludes causal inference.

Another important aspect of food environment perception concerns the price of foods, particularly healthy ones. Cost represents a major barrier to accessing these foods and ensuring the Human Right to Adequate Food (HRAF) [32,33]. During the pandemic, rising food prices were reported as an obstacle to purchasing food for 32.7% of participants in a survey conducted in 82 countries. Moreover, participants reported reduced variety (50.4%), quality (30.2%), and quantity (39.2%) of foods available during this period [29].

Affordability is a key factor in accessing healthy foods, particularly for low-income populations. Characteristics related to income, such as lower wages and income losses during the pandemic, were associated with greater household availability of UPF than FV. It is also important to highlight the high perceived ease of purchase and the variety of UPF, reflecting their widespread distribution and accessibility in neighbourhoods. This perception may be related to features of the local food environment, in which there may be a higher concentration of shops selling UPF and fewer—or even no—shops that sell FV. Overall, evidence indicates a scarcity of establishments selling healthy foods in socially vulnerable areas, hindering residents’ access to such items, particularly in peripheral regions with low per capita income [25,32,34,35].

Given the pandemic context, due to the suspension of municipal school activities and the temporary absence of school meals, some municipalities implemented food distribution programmes for families. In Mariana, monthly food baskets were distributed to families of students in the municipal network, funded by the National School Feeding Programme (PNAE). These baskets contained staple foods such as rice, beans, pasta, oil, coffee, flour, powdered milk, and UPF such as biscuits. In Ouro Preto, food kits containing vegetables from family-based agriculture were distributed, although delivery was irregular and did not cover all students. No distribution of high-biological-value protein sources, such as meat or eggs, was observed in either municipality [36].

Physical access to healthy foods alone may be insufficient to promote healthy eating, highlighting the importance of financial conditions and economic accessibility, particularly among low-income families. Regarding the food environment specifically, initiatives that facilitate physical and economic access to healthy foods are urgently needed, alongside strategies to reduce UPF availability. Taxation of UPF is one of the strategies recommended by the World Health Organisation [37] and has been adopted in several countries to reduce consumption of these products. However, strengthening the production and distribution of unprocessed foods, subsidising their production, and combining these actions with interventions that ensure physical and economic access to other basic, healthy foods are also essential. Examples include implementing facilities dedicated to selling FV, such as street markets, community gardens, and short supply chains [7].

In addition to policies targeting the food environment, social protection measures are crucial to reducing economic and social inequalities. The COVID-19 pandemic highlighted the importance of programmes that provide income support to vulnerable populations, such as Bolsa Família (2003–2021) and Auxílio Brasil (2021–2022), which were essential in mitigating the pandemic’s effects on families [38,39]. The literature suggests that such programmes should be maintained and expanded to ensure that families have access to quality food [40,41]. Furthermore, the National School Feeding Programme (PNAE) plays a key role in providing adequate and healthy meals for school-aged children, as meals are based on unprocessed and minimally processed foods such as FV, and the purchase of UPF is prohibited. For some students, particularly those in socioeconomically vulnerable situations, school meals are highly important, often representing their main daily meal [42,43].

Finally, due to the study’s cross-sectional design, causal relationships cannot be established. Although food environment perception may influence household food availability, it is also possible that food availability itself influences perception, suggesting potential endogeneity that cannot be formally tested. Additionally, a temporal misalignment exists between the perception and food availability measures: while perception questions referred to the current neighbourhood food environment, household food availability was assessed over the preceding 30 days. Although data collection occurred during a period of relative stability in the local food environment (between major COVID-19 pandemic waves in Brazil), the possibility that perceptions may not fully reflect conditions throughout the entire recall period cannot be excluded.

Regarding analytical decisions, the dichotomisation of the food availability scale into ‘low’ and ‘high’ categories may have resulted in some loss of information, as meaningful differences within intermediate frequency categories could not be captured. The causal framework underlying the analytical model was not formally specified, which limits the ability to fully distinguish confounders from potential mediators among the covariates included in the adjusted models. Additionally, the replacement of non-respondents through new random draws may have introduced selection bias, as households that were unreachable or unavailable may differ systematically from those included in the study. Furthermore, the household food availability questionnaire was adapted from a national study rather than formally validated, which may limit the precision of the availability estimates; however, the food items included were selected based on their relevance to the Brazilian diet, as documented in nationally representative dietary surveys. Therefore, the estimated associations should be interpreted as correlational rather than causal.

## 5. Conclusions

This study shows that perceptions of the food environment are associated with household UPF availability. A positive perception of FV variety in the neighbourhood was associated with lower odds of high UPF availability, while the perception of a wide variety of UPF in the neighbourhood was associated with higher odds of high UPF availability at home. These findings suggest that the neighbourhood food environment, as perceived by residents, may be relevant to household food availability patterns. In this context, policies aimed at promoting healthier food environments—such as expanding the supply and distribution of unprocessed foods and ensuring economic access to them—may be worth considering, though causal evidence from longitudinal studies is needed to support more definitive recommendations.

Despite these findings, this study has some limitations, such as the use of telephone interviews, which included only families with access to a mobile phone or an active landline, potentially leading to participation bias. This format was adopted due to the pandemic context during data collection; however, it is worth noting that approximately 96% of the Brazilian population owns a mobile phone for personal use, and 15.6% of Brazilian households have a landline [44]. In addition, only students enrolled in public schools were included; therefore, the findings represent students from the municipal public school network.

Future studies may explore other data collection methods, include students from private education networks, and investigate additional dimensions of the food environment, such as the presence of food stores in the neighbourhood. Longitudinal studies may also help clarify causal relationships between perceptions of the food environment and household food availability.

## Figures and Tables

**Table 1:** Sociodemographic characteristics of households according to the availability of unprocessed or minimally processed foods (NOVA Group 1) and ultra-processed foods (NOVA Group 4)—ESANP, 2021.

Variables	All (n = 475)	Availability of Unprocessed and Minimally Processed Foods		*p*-Value *	Availability of Ultra-Processed Foods		*p*-Value *
	% (95% CI)	% (95%CI)	% (95%CI)		% (95%CI)	% (95%CI)	
		Low (n = 440)	High (n = 35)		Low (n = 61)	High (n = 414)	
**Head of Household**							
**Sex**				0.224			**0.038**
Male	50.1 (45.6–54.6)	49.4 (44.6–53.9)	60.0 (42.9–74.9)		37.7 (26.3–50.5)	52.0 (47.0–56.7)	
Female	49.9 (45.3–54.3)	50.6 (46.0–55.3)	40.0 (44.6–53.9)		62.3 (49.4–73.6)	48.0 (43.2–52.9)	
**Referred Skin colour**				0.451			
White	19.2 (15.8–23.1)	18.8 (15.4–22.8)	24.2 (12.4–41.8)		19.4 (15.7–23.5)	18.3 (10.3–30.3)	0.846
Blacks (brown/black)	80.8 (76.8–84.1)	81.2 (77.1–84.5)	75.8 (58.1–84.5)		80.6 (76.4–84.2)	81.7 (69.6–89.6)	
**Age**				0.853			0.535
Between 18 and 35 years	30.1 (26.1–34.3)	29.8 (25.6–34.2)	34.3 (20.4–51.4)		37.7 (26.3–50.5)	29.0 (24.8–33.5)	
Between 36 and 45 years	40.4 (36.0–44.9)	40.6 (36.1–45.3)	37.2 (22.7–54.2)		34.4 (23.5–47.2)	41.3 (36.6–46.1)	
Between 46 and 60 years	23.2 (19.5–27.1)	23.5 (19.6–27.6)	20.0 (9.7–36.7)		23.0 (14.0–35.2)	23.2 (19.3–27.5)	
61 years or older	6.3 (4.4–8.9)	6.1 (4.2–8.8)	8.5 (2.7–23.8)		4.9 (1.5–14.3)	6.5 (4.5–9.3)	
**Educational level**				**0.013**			**0.008**
Elementary	29.3 (25.2–33.5)	30.3 (26.2–34.8)	14.8 (6.1–31.1)		42.7 (30.7–55.3)	27.3 (23.1–31.7)	
High school	60.4 (55.8–64.7)	60.3 (55.5–64.7)	61.7 (44.4–76.5)		55.7 (43.0–67.7)	61.1 (56.2–65.6)	
University	10.3 (7.9–13.4)	9.4 (6.9–12.4)	23.5 (12.0–40.7)		1.6 (0.2–10.9)	11.6 (8.9–15.1)	
**Marital status**				**0.029**			0.112
Married	61.6 (57.2–65.9)	59.5 (54.4–64.2)	72.3 (61.6–80.9)		52.4 (39.9–64.7)	63.1 (58.2–67.5)	
Not married	38.4 (34.0–42.7)	40.5 (35.7–45.5)	27.7 (19.0–38.3)		47.6 (35.2–60.6)	36.9 (32.4–41.7)	
**Household**							
**Family income**				**<0.001**			**<0.001**
Up to half the minimum wage	19.5 (16.2–23.4)	21.7 (17.8–26.0)	9.7 (4.8–18.2)		36.1 (24.9–48.8)	17.2 (13.8–21.1)	
1 to 2 minimum wages	63.2 (58.7–67.3)	64.3 (59.3–68.8)	57.8 (46.9–68.0)		62.3 (49.4–73.6)	63.2 (58.5–67.8)	
3 minimum wages or more	17.3 (14.1–20.9)	14.0 (10.9–17.8)	32.5 (23.2–43.3)		1.6 (0.2–10.8)	19.6 (16.0–23.6)	
**Benefits (social programmes)**				0.767			**0.008**
Yes	45.4 (40.9–49.8)	45.2 (40.2–50.1)	43.4 (33.0–54.2)		60.6 (47.8–72.1)	42.6 (37.8–47.3)	
No	54.6 (50.1–59.0)	54.8 (49.8–59.7)	56.6 (45.7–66.9)		39.4 (27.8–52.1)	57.4 (52.6–62.1)	
**Number of adults in the household**				0.251			0.096
1 Adult	15.8 (12.7–19.3)	08.6 (02.9–22.4)	16.4 (13.2–20.1)		24.6 (15.5–36.7)	14.5 (11.4–18.2)	
2 adults	55.8 (51.5–60.2)	68.6 (52.0–81.4)	54.8 (50.1–59.4)		45.9 (34.0–58.3)	57.2 (52.4–61.8)	
3 adults or more	28.4 (24.5–32.6)	22.9 (12.1–39.0)	28.9 (24.9–33.3)		29.5 (19.6–41.9)	28.3 (24.1–32.8)	
**Number of children in the household**				0.26			**0.048**
1 child	41.5 (37.1–45.9)	31.4 (18.6–48.0)	42.3 (37.7–46.9)		36.1 (25.2–48.6)	42.2 (37.6–47.1)	
2 children	41.7 (37.3–46.1)	42.9 (27.9–59.1)	41.6 (37.1–46.2)		36.1 (25.1–48.6)	42.6 (37.8–47.3)	
3 children or more	16.8 (13.7–20.4)	25.7 (14.2–42.1)	16.1 (13.0–19.9)		27.8 (18.2–40.2)	15.2 (12.1–19.0)	
**Received emergency financial aid**				**0.003**			0.064
Yes	27.8 (23.9–32.0)	30.6 (26.2–35.3)	14.4 (8.3–23.8)		37.7 (26.3–50.5)	26.4 (22.2–30.8)	
No	72.2 (67.9–76.0)	69.4 (64.6–73.7)	85.6 (76.1–91.6)		63.3 (49.4–73.6)	73.6 (69.1–77.7)	
**Fall in household income during the pandemic**				**0.002**			**0.002**
Yes	62.4 (57.8–66.5)	65.5 (60.6–70.1)	47.0 (36.4–57.7)		80.4 (68.3–88.5)	59.6 (54.8–64.3)	
No	37.6 (33.4–42.1)	34.5 (29.8–39.3)	53.0 (42.2–63.5)		19.6 (11.4–31.6)	40.4 (35.6–45.1)	
**Impact of the fall on household income**				**0.004**			**<0.001**
No impact	37.5 (33.4–42.1)	34.5 (29.8–39.3)	53.0 (42.2–63.5)		19.6 (11.4–31.6)	40.4 (35.6–45.1)	
Small	4.5 (2.8–6.6)	4.8 (3.1–7.4)	2.4 (5.9–9.2)		3.3 (0.1–12.3)	4.5 (2.9–7.0)	
Medium	21.5 (17.9–25.4)	21.2 (17.3–25.5)	22.9 (15.0–33.2)		18.0 (10.2–29.8)	22.0 (18.2–26.2)	
Large	36.5 (32.1–40.8)	39.5 (34.7–44.4)	21.7 (14.0–31.9)		59.1 (46.2–70.6)	33.1 (28.7–37.7)	

Note: The minimum wage at the time of the survey was US$ 207.27. * In bold: statistically significant associations according to Pearson’s chi-square test (*p*  <  0.05). 95%CI: 95% confidence interval.

**Table 2:** Perception of the food environment according to the total sample and household food availability—ESANP, 2021.

Variables		All (n = 475)	Availability of Unprocessed and Minimally Processed Foods		*p*-Value *	Availability of Ultra-Processed Foods		*p*-Value *
			Low (n = 440)	High (n = 35)		Low (n = 61)	High (n = 414)	
		% (95%CI)	% (95%CI)	% (95%CI)		% (95%CI)	% (95%CI)	
**It is easy to buy FV in the neighbourhood**	Agree	64.0 (59.5–68.2)	64.1 (59.4–68.4)	62.8 (45.7–77.2)	0.884	50.8 (38.3–63.1)	65.9 (61.2–70.3)	**0.022**
	Disagree	36.0 (31.7–40.4)	35.9 (31.5–40.5)	37.1 (22.7–54.2)		49.1 (36.8–61.6)	34.1 (29.6–38.7)	
**FV are of good quality in the neighbourhood**	Agree	67.6 (63.2–71.6)	67.0 (62.4–71.2)	74.2 (57.1–86.2)	0.378	54.1 (41.4–66.2)	69.5 (64.9–73.8)	**0.016**
	Disagree	32.4 (28.3–36.7)	33.0 (28.7–37.5)	25.8 (13.8–42.8)		45.9 (33.7–58.5)	30.5 (26.1–35.0)	
**There is a wide variety of FV in the neighbourhood**	Agree	57.9 (53.3–62.2)	57.7 (53.0–62.2)	60.0 (42.9–74.9)	0.793	40.9 (29.3–53.7)	60.3 (55.5–65.0)	**0.004**
	Disagree	42.1 (37.7–46.6)	42.3 (37.7–46.9)	40.0 (25.0–57.0)		59.1 (46.2–70.6)	39.7 (34.9–44.4)	
**FV are cheap in the neighbourhood**	Agree	20.5 (16.8–24.4)	19.8 (16.2–23.9)	28.1 (15.1–46.1)	0.261	15.2 (8.0–26.9)	21.3 (17.3–25.6)	0.291
	Disagree	79.5 (75.5–83.1)	80.2 (76.0–83.7)	71.9 (53.8–84.4)		84.8 (73.0–91.9)	78.7 (74.3–82.6)	
**It is easy to buy UPF in the neighbourhood**	Agree	85.9 (82.4–88.7)	85.5 (81.8–88.4)	91.5 (76.1–97.2)	0.328	78.7 (66.9–87.1)	86.9 (83.3–89.8)	0.083
	Disagree	14.1 (11.2–17.5)	14.5 (11.5–18.1)	8.5 (2.7–23.8)		21.3 (12.7–33.4)	13.1 (10.1–16.6)	
**There is a wide variety of UPF in the neighbourhood**	Agree	78.9 (75.0–82.3)	79.0 (75.0–82.6)	77.1 (60.2–88.2)	0.786	68.9 (56.1–79.2)	80.4 (76.3–83.9)	**0.038**
	Disagree	21.1 (17.6–24.9)	21.0 (17.3–24.9)	22.9 (11.7–39.7)		31.1 (20.7–43.8)	19.6 (16.0–23.6)	

Note: * In bold: statistically significant associations (*p* < 0.05). *p*-values correspond to Pearson’s chi-square tests comparing the distribution of perception variables between household food availability subgroups (low vs. high). 95%CI: 95% confidence interval; FV = fruits and vegetables; UPF = ultra-processed foods.

**Table 3:** Unadjusted and adjusted models of association between perceived food environment and household food availability—ESANP, 2021.

Perception of the Food Environment	Food Availability			
	Unprocessed and Minimally Processed Foods	Unprocessed and Minimally Processed Foods	Ultra-Processed Foods	Ultra-Processed Foods
	Unadjusted OR (95%CI)	Adjusted OR (95%CI)	Unadjusted OR (95%CI)	Adjusted OR (95%CI)
It is easy to buy FV in the neighbourhood ^a^	0.94 (0.46–1.93)	0.87 (0.40–1.90)	**0.53 (0.31–0.91)**	0.65 (0.34–1.09)
FV are of good quality in the neighbourhood ^a^	1.41 (0.64–3.10)	1.25 (0.52–3.01)	**0.51 (0.29–0.88)**	0.63 (0.35–1.12)
There is a wide variety of FV in the neighbourhood ^a^	1.09 (0.54–2.21)	1.13 (0.52–2.48)	**0.45 (0.26–0.78)**	**0.54 (0.30–0.97)**
FV are cheap in the neighbourhood ^a^	1.58 (0.70–3.55)	1.74 (0.72–4.21)	0.66 (0.31–1.41)	0.77 (0.35–1.71)
It is easy to buy UPF in the neighbourhood ^b^	0.55 (0.16–1.85)	0.47 (0.12–1.75)	1.80 (0.91–3.55)	1.84 (0.89–3.82)
There is a wide variety of UPF in the neighbourhood ^b^	1.12 (0.49–2.54)	1.08 (0.41–2.81)	**1.85 (1.02–3.36)**	1.54 (0.81–2.93)

Note: In bold: statistically significant associations (*p* < 0.05). OR = odds ratio; CI = confidence interval. Unadjusted models represent unadjusted logistic regression analyses. Adjusted models were controlled for decrease in family income during the pandemic, sex and educational level of the head of household, marital status, participation in social programmes, receipt of emergency financial aid, and number of children in the household. ^a^ Reference category for perception of FV: agree. ^b^ Reference category for perception of UPF: disagree. Reference category for the outcome: high FV availability at home; low UPF availability at home. FV = fruits and vegetables; UPF = ultra-processed foods.

## Data Availability

The data presented in this study are available on request from the corresponding author.

## References

[1] FAO, IFAD, UNICEF, WFP, WHO (2020). The State of Food Security and Nutrition in the World 2020. Transforming Food Systems for Afordable Healthy Diets.

[2] High Level Panel of Experts (2020). Food Security and Nutrition: Building a Global Narrative Towards 2030. A Report by the High-Level Panel of Experts on Food Security and Nutrition of the Committee on World Food Security.

[3] Benites-Zapata V.A., Urrunaga-Pastor D., Solorzano-Vargas M.L., Herrera-Añazco P., Uyen-Cateriano A., Bendezu-Quispe G., Toro-Huamanchumo C.J., Hernandez A.V. (2021). Prevalence and factors associated with food insecurity in Latin America and the Caribbean during the first wave of the COVID-19 pandemic. Heliyon.

[4] Ogundijo D.A., Tas A.A., Onarinde B.A. (2021). Exploring the Impact of COVID-19 Pandemic on Eating and Purchasing Behaviours of People Living in England. Nutrients.

[5] Andrade G.C., Caldeira T.C.M., Mais L.A., Martins A.P.B., Claro R.M. (2024). Food price trends during the COVID-19 pandemic in Brazil. PLoS ONE.

[6] Lourenço A.E.P., Sperandio N., Pontes P.V., Monteiro L.S. (2021). School Feeding and Food and Nutrition Security in the Context of the Covid-19 Pandemic in the Northern Region of the State of Rio de Janeiro, Brazil. Food Ethics.

[7] Rodrigues E.C. (2023). Determinantes Sociodemográficos da Insegurança Alimentar nos Domicílios de Estudantes de Escolas Públicas: Uma Investigação Longitudinal.

[8] Araújo M.L., Mendonça R.D., Lopes Filho J.D., Lopes A.C.S. (2018). Association between food insecurity and food intake. Nutrition.

[9] Elsahoryi N., Al-Sayyed H., Odeh M., McGrattan A., Hammad F. (2020). Effect of Covid-19 on food security: A cross-sectional survey. Clin. Nutr. ESPEN.

[10] Leone L.A., Fleischhacker S., Anderson-Steeves B., Harper K., Winkler M., Racine E., Baquero B., Gittelsohn J. (2020). Healthy Food Retail during the COVID-19 Pandemic: Challenges and Future Directions. Int. J. Environ. Res. Public Health.

[11] Maia E.G., dos Passos C.M., Levy R.B., Martins A.P.B., Mais L.A., Claro R.M. (2020). What to expect from the price of healthy and unhealthy foods over time? The case from Brazil. Public Health Nutr..

[12] Story M., Kaphingst K.M., Robinson-O’Brien R., Glanz K. (2008). Creating Healthy Food and Eating Environments: Policy and Environmental Approaches. Annu. Rev. Public Health.

[13] Glanz K., Handy S.L., Henderson K.E., Slater S.J., Davis E.L., Powell L.M. (2016). Built environment assessment: Multidisciplinary perspectives. SSM Popul. Health.

[14] Pérez E., Roncarolo F., Potvin L. (2017). Associations between the local food environment and the severity of food insecurity among new families using community food security interventions in Montreal. Can. J. Public Health.

[15] Menezes M.C., Diez Roux A.V., Costa B.V.L., Lopes A.C.S. (2018). Individual and food environmental factors: Association with diet. Public Health Nutr..

[16] Yamaguchi M., Praditsorn P., Purnamasari S.D., Sranacharoenpong K., Arai Y., Sundermeir S.M., Gittelsohn J., Hadi H., Nishi N. (2022). Measures of Perceived Neighborhood Food Environments and Dietary Habits: A Systematic Review of Methods and Associations. Nutrients.

[17] Brazilian Institute of Geography and Statistics (2024). IBGE Cidades. https://cidades.ibge.gov.br/brasil/mg/ouro-preto/panorama.

[18] Brazilian Institute of Geography and Statistics (2020). Household Budget Survey 2017–2018: Analysis of Food Security in Brazil.

[19] Ministério da Saúde (2020). Vigitel Brasil 2019: Vigilância de Fatores de Risco e Proteção para Doenças Crônicas por Inquérito Telefônico.

[20] Dias dos Santos C., Rodrigues É., Camargo P., Justiniano I., de Carvalho N., de Menezes M., Meireles A., de Mendonça R. (2023). Disponibilidade, acesso percebido e insegurança alimentar em domicílios de escolares de dois municípios de Minas Gerais na pandemia de COVID-19. Segur. Aliment. Nutr..

[21] National Study of Infant Feeding and Nutrition. ENANI. https://enani.nutricao.ufrj.br.

[22] Avelar B.A., Hino A.A.F., Santos A.P., Mendes L.L., Carraro J.C.C., Mendonça R.d.D., de Menezes M.C. (2023). Validity and reliability of the Perceived Nutrition Environment Measures Survey (NEMS-P) for use in Brazil. Public Health Nutr..

[23] Monteiro C.A., Ricardo C.Z., Calixto G., Machado P., Martins C., Steele E.M., Baraldi L.G., Garzillo J.M.F., Sattamini I., Cannon G. (2016). NOVA The star shines bright. World Nutr..

[24] Ministry of Health (2014). Dietary Guidelines for the Brazilian Population.

[25] Rocha L.L., Friche A.A.d.L., Jardim M.Z., Junior P.C.P.d.C., Oliveira E.P., Cardoso L.d.O., Mendes L.L. (2024). Percepção dos residentes de favelas brasileiras sobre o ambiente alimentar: Um estudo qualitativo. Cad. Saude Publica.

[26] Caspi C.E., Sorensen G., Subramanian S., Kawachi I. (2012). The local food environment and diet: A systematic review. Health Place.

[27] Duran A.C., de Almeida S.L., Latorre M.D.R.D., Jaime P.C. (2015). The role of the local retail food environment in fruit, vegetable and sugar-sweetened beverage consumption in Brazil. Public Health Nutr..

[28] Costa B.V.L., Menezes M.C., Oliveira C.D.L., Mingoti S.A., Jaime P.C., Caiaffa W.T., Lopes A.C.S. (2019). Does access to healthy food vary according to socioeconomic status and to food store type? an ecologic study. BMC Public Health.

[29] Jafri A., Mathe N., Aglago E.K., O Konyole S., Ouedraogo M., Audain K., Zongo U., Laar A.K., Johnson J., Sanou D. (2021). Food availability, accessibility and dietary practices during the COVID-19 pandemic: A multi-country survey. Public Health Nutr..

[30] Barbosa B.B., Nielsen L., de Aguiar B.S., Failla M.A., Araújo L.F., Mendes L.L., Machado S.P., Carioca A.A.F. (2023). Local Food Environment and Consumption of Ultra-Processed Foods: Cross-Sectional Data from the Nutritionists’ Health Study—NutriHS. Int. J. Environ. Res. Public Health.

[31] Oladele C.R., Colón-Ramos U., Galusha D., Tran E., Adams O.P., Maharaj R.G., Nazario C.M., Nunez M., Pérez-Escamilla R., Nunez-Smith M. (2022). Perceptions of the local food environment and fruit and vegetable intake in the Eastern Caribbean Health Outcomes Research Network (ECHORN) Cohort study. Prev. Med. Rep..

[32] de Araújo M.L., Silva G.B., Rocha L.L., Novaes T.G., de Lima C.A.M., Mendes L.L., Pessoa M.C. (2022). Características do ambiente alimentar comunitário e do entorno das residências das famílias beneficiárias do Programa Bolsa Família. Cienc. Saude Coletiva.

[33] Skalkos D., Kalyva Z.C. (2023). Exploring the Impact of COVID-19 Pandemic on Food Choice Motives: A Systematic Review. Sustainability.

[34] Justiniano I.C.S., de Menezes M.C., Mendes L.L., Pessoa M.C. (2022). Retail food environment in a Brazilian metropolis over the course of a decade: Evidence of restricted availability of healthy foods. Public Health Nutr..

[35] Rocha L.L., Carmo A.S.D., Jardim M.Z., Leme B.A., Cardoso L.d.O., Caiaffa W.T., Andrade A.C.d.S., dos Santos L.C., Mendes L.L. (2021). The community food environment of a Brazilian metropolis. Food Cult. Soc..

[36] Rodrigues E.C., Mendonça R.d.D., Camargo P.P., de Menezes M.C., de Carvalho N.C., Meireles A.L. (2022). Home food insecurity during the suspension of classes in Brazilian public schools due to the COVID-19 pandemic. Nutrition.

[37] World Health Organization (WHO) (2016). Fiscal Policies for Diet and the Prevention of Noncommunicable Diseases: Technical Meeting Report, 5–6 May 2015, Geneva, Switzerland.

[38] Cardoso D.F., Domingues E., Magalhães A., Simonato T., Miyajima D. (2021). COVID-19 pandemic and families: Impacts of the crisis and the emergency basic income. Soc. Policies Monit. Anal..

[39] Costa D.M., Magalhães R., Cardoso M.L.d.M. (2023). From Bolsa Família to Auxílio Brasil: Challenges and achievements based on a program theory-based evaluative research. Cad. Saúde Pública.

[40] Pereira M., Oliveira A.M. (2020). Poverty and food insecurity may increase as the threat of COVID-19 spreads. Public Health Nutr..

[41] Ribeiro-Silva R.d.C., Pereira M., Campello T., Aragão É., Guimarães J.M.d.M., Ferreira A.J., Barreto M.L., dos Santos S.M.C. (2020). Implications of the COVID-19 pandemic for food and nutrition security in Brazil. Cienc. Saude Coletiva.

[42] National Fund for Educational Development (FNDE) (2020). Resolução Nº 6 de 08 de Maio de 2020.

[43] Mota C.H., de Barros Silva Mastroeni S.S., Mastroeni M.F. (2013). Consumo da refeição escolar na rede pública municipal de ensino. Rev. Bras. Estud. Pedagog..

[44] Brazilian Institute of Geography and Statistics. IBGE (2022). Continuous National Household Sample Survey.

